# Performance of Simple Outpatient-based Biomarker Panels for Screening of Steatotic Liver Disease in Women with Morbid Obesity from Southern India

**DOI:** 10.17925/EE.2025.21.1.3

**Published:** 2025-03-05

**Authors:** Shubhashis Saha, Shaleen Dass, Kripa Elizabeth Cherian, Stephen A Jiwanmall, Dheeraj Kattula, Sandhiya Reddy, Rajeeb Jaleel, Thomas V Paul, Nitin Kapoor

**Affiliations:** 1. Department of Endocrinology, Diabetes and Metabolism, Christian Medical College, Vellore, India; 2. Department of Psychiatry, Christian Medical College, Vellore, India; 3. Department of Gastroenterology, Christian Medical College, Vellore, India; SS and SD contributed equally.

**Keywords:** Biomarker, metabolic dysfunction-associated steatotic liver disease (MASLD), morbid obesity, non-alcoholic fatty liver disease (NAFLD), screening, women

## Abstract

**Background.:**

Non-alcoholic fatty liver disease (NAFLD) and related health issues are increasing in Indian women with morbid obesity, but the standard diagnostic tool – abdominal ultrasound sonography (USG) – is costly and less accessible. This study aims to identify an affordable and effective biomarker panel to improve early detection and screening of NAFLD in resource-l imited settings.

**Methods.:**

This cross-sectional study included 106 consecutive patients aged between 18 and 70 years with morbid obesity defined by body mass index (BMI) ≥32.5 kg/m^2^ who underwent an abdominal USG for a non-hepatic indication. The serum biomarker indices used were hepatic steatosis index, lipid accumulation product (LAP), Framingham steatosis index, triglyceride-glucose (TyG) index, TyG weight-to-height ratio composite index, TyG-BMI and TyG waist circumference composite index.

**Results.:**

The mean age was 40.2 ± 10.9 years, and the mean BMI was 41.5 ± 5.8 kg/m^2^. NAFLD was diagnosed in 71.7% of the participants. The TyG index showed the highest diagnostic accuracy with an area under the receiver operating characteristic curve (AUROC) of 0.835 (confidence interval [CI]: 0.713–0.957, p<0.001), with a sensitivity of 95.1% and a specificity of 70.8% at a cut-off of 9.0994. LAP showed an AUROC of 0.711 (CI: 0.584–0.838, p-value: 0.002). Using a cut-off score of 76.2, the sensitivity and specificity were 71.2 and 70.8%, respectively.

**Conclusion.:**

Simple screening tools can be used to detect fatty liver disease in clinical practice. In our cohort, TyG index was found to be the best tool for identifying NAFLD, with LAP showing potential as a secondary option.

## Article Highlights

- This study aims to find an effective and affordable biomarker panel for early non-alcoholic fatty liver disease (NAFLD) detection in Indian women with morbid obesity, given the rising prevalence of NAFLD and limited accessibility of ultrasound sonography (USG) for diagnosis in resource-l imited settings.
- The study included 106 women with morbid obesity who underwent an abdominal USG, at a single tertiary care centre in southern India.
- The triglyceride-glucose (TyG) index showed an area under the receiver operating characteristic curve (AUROC) of 0.835 with a sensitivity and specificity of 95.1 and 70.8%, respectively. Lipid accumulation product (LAP) showed an AUROC of 0.711, with a sensitivity and specificity of 71.2 and 70.8%, respectively.
- TyG weight-to-height ratio composite index had an AUROC of 0.752, showing good diagnostic ability, while TyG waist circumference composite index had an AUROC of 0.720. TyG-body mass index had the lowest AUROC of 0.638 but remained statistically significant.
- Framingham steatosis index and hepatic steatosis index were found to have indeterminate results.
- TyG index is a promising biomarker for screening NAFLD in women with morbid obesity and can be a useful tool for identifying patients who may require further testing for NAFLD, while LAP also shows promise as a secondary option.

Non-alcoholic fatty liver disease (NAFLD) is one of the hepatic manifestations of metabolic syndrome, strongly associated with obesity. The worldwide prevalence of NAFLD has been reported to be 32.4% (95% confidence interval [CI]: 29.9–34.9%) according to meta-analysis.^1^ In a study by Atri et al., on 106 consecutive morbidly obese women, the prevalence of NAFLD among the study population was found to be more than twice the proportion at 73.6% (95% CI: 65.2–82.0).^2^

Liver biopsy is considered the gold standard for diagnosing NAFLD, but it has limitations such as high cost, invasiveness and sampling variability. To exclude fibrosis in patients with NAFLD, scores like Fibrosis-4 (FIB-4) and NAFLD-fibrosis are used, and magnetic resonance imaging (MRI) is recommended to accurately quantify steatosis and identify fibrosis. However, implementing MRI in routine practice, especially in resource-l imited settings, can be challenging.^3^

Given these challenges, this study aims to evaluate the diagnostic accuracy of non-i nvasive indices such as the triglyceride-glucose (TyG) index, lipid accumulation product (LAP) and hepatic steatosis index (HSI) for the detection of NAFLD in a population of morbidly obese women. Furthermore, we aim to compare the effectiveness of these indices with standard diagnostic tools like liver ultrasound sonography (USG) in identifying NAFLD and to assess their potential as affordable, practical screening tools for use in secondary healthcare settings, particularly in low-i ncome regions.

At our secondary hospital under the Department of Community Health and Development, Christian Medical College Vellore, a liver USG costs 1,505 Indian rupees (INR), and a liver biopsy is 2,415 INR. In contrast, the LAP index requires only a triglyceride test costing 365 INR, while the TyG index requires fasting sugar and triglyceride tests for a total of 520 INR, and the HSI using the alanine transaminase (ALT)/aspartate transaminase (AST) ratio costs 540 INR. These affordable, non-i nvasive indices could offer a practical initial screening solution for hepatic steatosis in resource-l imited settings.

NAFLD is closely intertwined with obesity and type 2 diabetes (T2D), arising from insulin resistance-driven hepatic lipid accumulation.^4^ This process triggers oxidative stress, inflammation and intricate interactions between genetics and the gut microbiome. As NAFLD advances through stages of steatosis, non-alcoholic steatohepatitis (NASH), fibrosis and cirrhosis, its association with cardiovascular disease and chronic kidney disease (CKD) increases and so does morality, including that from a significantly increased incidence of hepatocellular carcinoma (HCC).

The severity of insulin resistance fuels the progression of NAFLD towards NASH and cirrhosis, creating a self-perpetuating cycle.^5^ The consequences include cirrhosis and HCC, where cirrhosis-associated fibrotic lesions and scar tissue impair liver function. Inflammation due to lipotoxicity exacerbates cellular hepatic injury, further impacting bilirubin metabolism. HCC, a severe outcome, can arise with or without cirrhosis and remains a significant cause of cancer. Obesity and diabetes play substantial roles in promoting HCC among patients with NAFLD.

Cardiovascular disease emerges as a pressing concern in NAFLD, as atherosclerosis markers intricately connect with NAFLD. Additionally, heart failure and CKD are associated with NAFLD. Extensive research covering over 10 million patients underscores the link between NAFLD and a heightened risk of all-cause mortality, cardiovascular disease-related mortality and cancer-related mortality. These findings, supported by multiple studies, accentuate the need for multidisciplinary strategies to address the broader spectrum of mortality risks posed by NAFLD, extending beyond liver-related concerns.^6,7^

This study will evaluate whether these non-i nvasive indices can serve as cost-effective tools to facilitate early diagnosis, helping to prevent disease progression and reduce associated mortality from cardiovascular disease, CKD and HCC.

## Methodology

### Study population and setting

This retrospective cross-sectional study was conducted in the Department of Endocrinology at Christian Medical College Vellore, a tertiary referral centre in Tamil Nadu, India. Ethical committee approval was obtained from the Institutional Review Board (IRB) of Christian Medical College Vellore (IRB min no. 10146). The research was conducted in accordance with the principles outlined in the Helsinki Declaration of 1964 and its later amendments. The study population comprised women with morbid obesity who presented to the bariatric clinic of our department in 2018. We chose to include only women in the study as they are the most commonly affected subgroup in the South Asian population.

We selected the HSI, TyG, LAP and Framingham steatosis index (FSI) among the commonly used serum biomarker indices for screening of NAFLD for our study based on the ease of calculating such indices in an outpatient setting regularly.

### Inclusion and exclusion criteria

We included all patients aged between 18 and 70 years with a body mass index (BMI) ≥32.5 kg/m^2^. We excluded all patients with a significant alcohol consumption history. All consecutive patients who met the inclusion criteria, were not excluded by the exclusion criteria and who underwent an abdominal USG for a non-hepatic indication were included in the study.

### Parameters used

Anthropometric data, including BMI and waist circumference, were collected from all patients per routine clinical protocol. The laboratory tests used by the indices were fasting serum triglyceride, fasting sugars and the ratio between AST and ALT, both of which were part of routine laboratory investigations performed for all new patients with morbid obesity as a standard of care. The USG reports of the included patients were used as the standard for the diagnosis of NAFLD. The formulae used for the calculation of the biomarker panels are provided in *Table 1*.

### Data collection and statistical analysis

With permission from the IRB, anonymized data were retrieved from our institution’s centralized clinical workstation portal. Before analysis, the data set was checked for missing data and outliers. The data were analysed using SPSS software (IBM Corp.; Released 2015; IBM SPSS Statistics for Windows, Version 23.0; Armonk, NY: IBM Corp.). Continuous variables are represented as means with standard deviations. Categorical data are represented by percentages. Receiver operating characteristic (ROC) curves were drawn for the three NAFLD biomarker panels. Optimal sensitivities and specificities for each index were obtained using the best-fit method for the highest Youden index for each ROC curve.

## Results

We included 106 women with morbid obesity, with a mean age of 40.2 ± 10.9 years. Among the anthropometric measures, the study population had a mean BMI of 41.5 ± 5.8 kg/m^2^, a mean height of 155.6 ± 6.7 cm, a mean weight of 155.6 ± 6.7 kg, a mean waist circumference of 117.7 ± 14.2 cm and a mean hip circumference of 126.1 ± 13.4 cm. The percentage of patients with NAFLD was 71.7%, and systemic hypertension, T2D and dyslipidaemia were present in 46.2% (95% CI: 36.7–55.8%), 38.7% (95% CI: 31.3–50.7%) and 47.2% (95% CI: 37.6–56.7%), respectively (*Table 2*).

**Table 1:** Calculation of the indices

Index	Formula
LAP	Waist circumference (cm)-58×triglycerides (mmol/L)
Framingham steatosis index	-7.981+0.011×age (years)-0.146×sex (female=1, male=0)+0.173×BMI (kg/m^2^)+0.007×triglycerides (mg/dL)+0.593×hypertension (yes=1, no=0)+0.789×diabetes (yes=1, no=0)+1.1×ALT/AST ratio ≥1.33 (yes=1, no=0)
Hepatic steatosis index	8×(ALT/AST ratio)+BMI (+2, if female;+2, if diabetes)
TyG index	ln(fasting triglyceride [mg/dL]×fasting glucose [mg/dL]/2)
TyG-WHtR	TyG×WHtR
TyG BMI	TyG index×BMI
TyG WC	TyG×WC

*ALT = alanine transaminase; AST = aspartate transaminase; BMI = body mass index; LAP = lipid accumulation product; TyG = triglyceride-glucose; TyG-WC = triglyceride-glucose and waist circumference composite index; TyG-WHtR = triglyceride-glucose and weight-to-height ratio composite index.*

When comparing patients with NAFLD (76, 71.7%) with those without (30, 28.3%), we found that fasting serum triglyceride was higher in the NAFLD group (147.3 ± 70.0 mg/dL) when compared with the non-NAFLD group (116.3 ± 59.7 mg/dL; p=0.043). Fasting serum total cholesterol was 168.2 ± 38.5 mg/dL in the former group compared with 151.9 ± 34.8 mg/dL in the latter group (p=0.063). Serum AST was 27.0 ± 18.1 mg/dL in the former group compared with 19.3 ± 6.7 in the latter group (p=0.019). Serum ALT was 26.9 ± 20.8 mg/dL in the former group compared with 20.1 ± 9.0 in the latter group (p=0.082). The serum AST:ALT ratio was 1.03 ± 0.44 in the former group compared with 0.95 ± 0.32 in the latter group (p=0.440). The above comparison is shown in *Table 3*.

**Figure 1:**
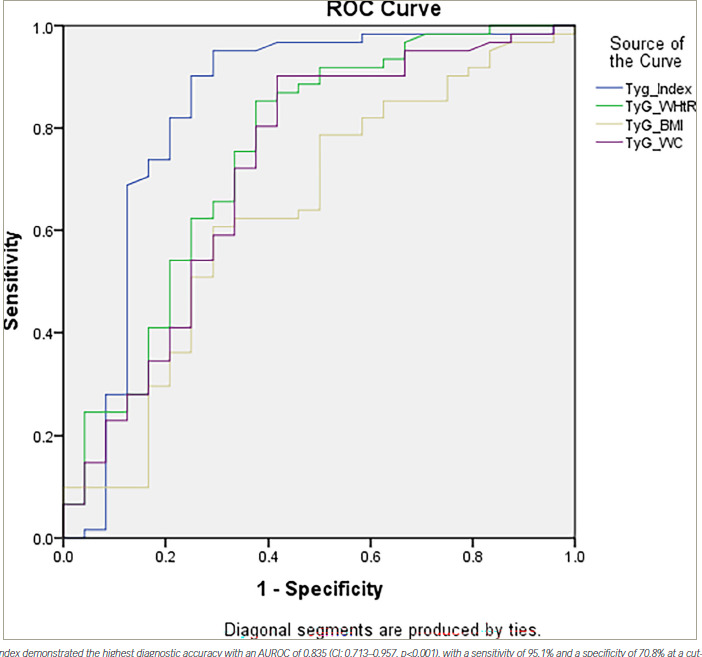
Receiver operating characteristic curve for the different screening indices

In our study, we assessed the efficacy of several indices in detecting NAFLD. The TyG index demonstrated a robust performance with an area under the ROC curve (AUROC) of 0.835 (95% CI: 0.713–0.957; p<0.001; *Figure 1*). Using the point on the ROC curve corresponding to the highest Youden index (calculated as sensitivity+specificity-1), we identified an optimal cut-off value of 9.0994 for the TyG index. This yielded a sensitivity of 95.1% and a specificity of 70.8%.

**Figure 2:**
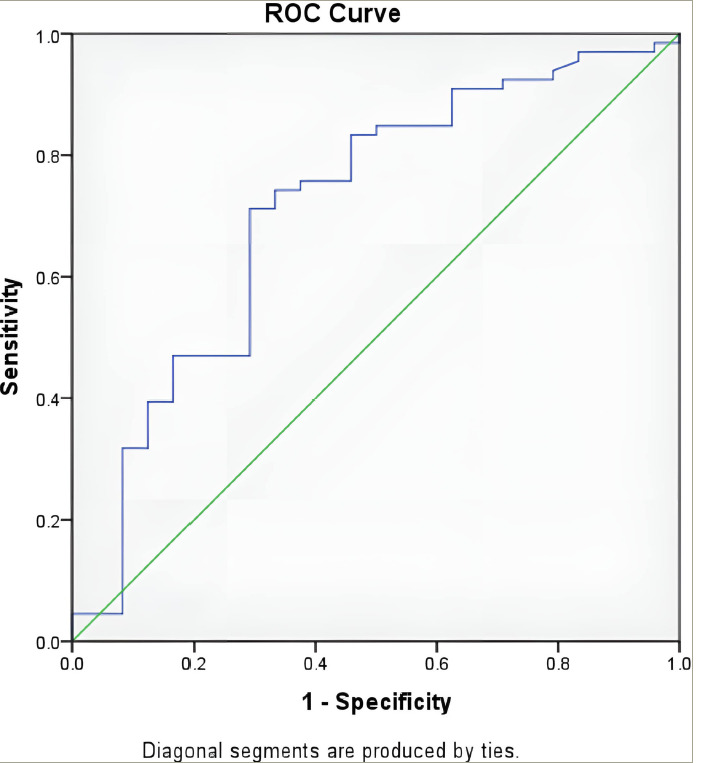
Receiver operating characteristic curve for the lipid accumulation product score

The LAP also showed significant predictive ability with an AUROC of 0.711 (95% CI: 0.584–0.838; p=0.002; *Figure 2*). The optimal cut-off for LAP was determined to be 76.2, resulting in a sensitivity of 71.2% and a specificity of 70.8%.

**Table 2:** Baseline characteristics

Baseline characteristics	Measure (95% CI)
Age at presentation (years) (mean ± SD)	40.2 ± 10.97 (38.1–42.3)
BMI (kg/m^2^) (mean ± SD)	41.6 ± 5.9 (40.48–42.7)
Weight (kg) (mean ± SD)	101.3 ± 16.9 (98.1–104.5)
Height (cm) (mean ± SD)	155.7 ± 6.72 (154.3–157)
Waist circumference (cm) (mean ± SD)	117.7 ± 14.20 (115–120)
Hip circumference (cm) (mean ± SD)	126.1 ± 13.40 (123.5–128.7)
Systemic hypertension (n, %)	49, 46.2% (0.3–0.6)
Type 2 diabetes (n, %)	41, 38.7% (0.3–0.5)
Dyslipidaemia (n, %)	50, 47.2% (0.4–0.6)
NAFLD (n, %)	76, 71.7% (0.2–0.4)

*BMI = body mass index; CI = confidence interval; NAFLD = non-alcoholic fatty liver disease.*

Conversely, FSI and HSI did not exhibit significant discriminatory power in our cohort. The AUROC for FSI was 0.536 (p=0.987), and for HSI, it was 0.525 (p=0.765), indicating no better performance than chance (*Table 4*). This suggests that FSI and HSI are indeterminate for diagnosing NAFLD in our population.

## Discussion

This study aims to evaluate the efficacy of various serum biomarker panels for NAFLD screening in women with morbid obesity in South India. The study compared the performance of the different biomarker panels with USG using ROC curves and calculated the area under the curve to analyse the effectiveness of each index.

NAFLD is a growing health concern, especially in developing countries due to urbanization and lifestyle changes. Our study supports the use of non-i nvasive indices like the HSI and LAP for diagnosing NAFLD in resource-l imited settings.

In a study done in India, it was reported that there was a high prevalence of NAFLD among non-obese individuals, suggesting that factors beyond obesity contribute significantly to hepatic steatosis.^8^ These findings highlight the need for accessible screening tools.

A study done in Brazil demonstrated a high prevalence of NAFLD in Brazilian patients with type 2 diabetes, reinforcing the association between NAFLD and metabolic disorders.^9^ Similarly, a study done in China found that TyG index-related parameters are effective for early diagnosis of NAFLD and liver fibrosis.^10^ Their study supports the utility of simple, non-i nvasive indices in early detection.

LAP has been advocated as a simple clinical indicator of NASH and metabolic syndrome, but not many studies have been done to establish its performance in detecting NAFLD in the South Asian population. Initially, it was established as a superior clinical indicator of metabolic syndrome compared with other measures of adiposity for the detection of metabolic syndrome in the Taiwanese population.^4^

The TyG index has emerged as a simple clinical indicator of insulin resistance and metabolic disorders, including NAFLD. In a cross-sectional study involving Chinese adults, the TyG index was found to be independently associated with NAFLD, even after adjusting for potential confounding factors. The study demonstrated that the TyG index was significantly higher in participants with NAFLD, indicating its potential utility as a non-i nvasive marker for identifying individuals at high risk of NAFLD.^10^

**Table 3:** Metabolic lab parameters

	Patients with NAFLD (n=76) (mean ± SD)	Patients without NAFLD (n=40) (mean ± SD)	p-value
Fasting serum triglyceride (mg/dL)	147.4 ± 70	116.4 ± 59.8	0.043
Fasting serum total cholesterol (mg/dL)	168.3 ± 38.6	151.9 ± 34.8	0.063
Fasting serum (mg/dL)	40.5 ± 11	40.9 ± 12.5	0.893
Fasting serum low-density lipoprotein (mg/dL)	109.3 ± 31.4	94.2 ± 29.1	0.039
Serum AST (mg/dL)	27 ± 18.1	19.31 ± 6.8	0.019
Serum ALT (mg/dL)	26.98 ± 20.9	20.18 ± 9	0.082
Serum AST:ALT ratio	1 ± 0.4	0.95 ± 0.3	0.440
HbA1c (%)	6.6 ± 1	6.6 ± 1.7	0.847
Systemic hypertension (n, %)	36, 48 %	13,43.3	0.670
Type 2 diabetes (n, %)	32, 42.7%	9, 30%	0.236
Dyslipidaemia (n, %)	38, 50.7 %	11, 36.7%	0.195

*ALT = alanine transaminase; AST = aspartate transaminase; HbA1c = haemoglobin A1c; NAFLD = non-alcoholic fatty liver disease.*

*The TyG index demonstrated the highest diagnostic accuracy with an AUROC of 0.835 (CI: 0.713–0.957, p<0.001), with a sensitivity of 95.1% and a specificity of 70.8% at a cut-off of 9.0994. In comparison, TyG-WHtR had an AUROC of 0.752, TyG-WC had 0.720 and TyG-BMI showed the lowest diagnostic performance with an AUROC of 0.638.*

*AUROC = area under the receiver operating characteristic curve; BMI = body mass index; CI = confidence interval; ROC = receiver operating characteristic; TyG = triglyceride-glucose; TyG-WC = triglyceride-glucose and waist circumference composite; TyG-WHtR = triglyceride-glucose and weight-to-height ratio composite.*

*The* LAP *score achieved an AUC of 0.711 with an optimal cut-off value of 76.2, yielding a sensitivity of 71.2% and a specificity of 70.8%. AUC = area under the curve; LAP = lipid accumulation product; ROC = receiver operating curve.*

HSI and FSI are also simple clinical indicators for NASH. They show promise as clinical indicators that may be used along with liver USG for selected individuals.^5^ The utility of FSI for predicting the risk of NAFLD in the Iranian population was evaluated in a study, and it was shown that FSI had a strong ability to diagnose NAFLD and an acceptable ability to predict the occurrence of new cases.^6^

In this study, a comparison was made between patients with NAFLD (76, 71.7%) and those without (40, 28.3%). The NAFLD group had higher levels of fasting serum triglycerides (mean value: 147.3 ± 70.0 mmol/dL) compared with the non-NAFLD group (mean value: 116.3 ± 59.7 mmol/dL) with a p-value of 0.043. Similarly, fasting serum total cholesterol levels were also higher in the NAFLD group (mean value: 168.2 ± 38.5 mmol/dL) compared with the non-NAFLD group (mean value: 151.9 ± 34.8 mmol/dL) with a p-value of 0.063. Additionally, serum AST levels were higher in the NAFLD group (mean value: 27.0 ± 18.1 mmol/dL) compared with the non-NAFLD group (mean value: 19.3 ± 6.7 mmol/dL) with a p-value of 0.019. The serum ALT levels showed a trend towards being higher in the NAFLD group (mean value: 26.9 ± 20.8 mmol/dL) compared with the non-NAFLD group (mean value: 20.1 ± 9.0 mmol/dL) with a p-value of 0.082. However, the serum AST:ALT ratio did not show any significant difference between the two groups, with a mean value of 1.03 ± 0.44 in the NAFLD group compared with 0.95 ± 0.32 in the non-NAFLD group (p=0.440).

In comparison, a similar study done in Chinese women showed a mean fasting serum triglyceride level of 159.43 ± 124 mmol/dL in the NAFLD group and 88.7 ± 53.14 mmol/dL in the non-NAFLD group, a mean fasting serum total cholesterol level of 208.82 ± 88.7 mmol/dL in the NAFLD group and 189.48 ± 34.8 mmol/dL in the non-NAFLD group and a serum ALT mean of 26.6 ± 18.6 mmol/dL in the NAFLD group and 17.5 ± 11.5 mmol/dL in the non-NAFLD group.^7^

**Table 4:** Receiver operating characteristic curves and their statistics parameters for lipid accumulation product, Framingham steatosis index and hepatic steatosis index

Biomarker panel	AUROC	Standard deviation	95 % CI Lower	Upper	p-value	Sensitivity (%)	Specificity (%)
LAP	0.711	0.065	0.584	0.838	0.002	71.2	70.8
FSI	0.536	0.088	0.364	0.708	0.676	61.4	43.8
HSI	0.525	0.077	0.373	0.676	0.765	60.4	39.6
TyG index	0.835	0.062	0.713	0.957	<0.001	95.1	70.8
TyG -WHtR	0.752	0.064	0.626	0.878	<0.001	85.2	62.5
TyG -BMI	0.638	0.7	0.501	0.776	0.048	60.7	70.8
TyG -WC	0.72	0.067	0.588	0.852	0.002	90.2	58.3

*AUROC = area under the receiver operating characteristic curve; BMI = body mass index; CI = confidence interval; FSI = Framingham steatosis index; HSI = hepatic steatosis index; LAP = lipid accumulation product; TyG = triglyceride-glucose; TyG-BMI = triglyceride-glucose and body mass index composite index; TyG-WC = triglyceride-glucose and waist circumference composite index; TyG-WHtR = triglyceride-glucose and weight-to-height ratio composite Index.*

Using ROC curve analysis, we evaluated the accuracy of several indices – HSI, FSI, LAP, TyG index, TyG and weight-to-height ratio composite index (TyG-WHtR), TyG-BMI and TyG and waist circumference composite index (TyG-WC) – for diagnosing NAFLD in women. Among these, the TyG index demonstrated the highest diagnostic performance, with an AUROC of 0.835 (95% CI: 0.713–0.957; p<0.001). At an optimal cut-off value of 9.0994, determined using the highest Youden index, the TyG index yielded a sensitivity of 95.1% and a specificity of 70.8%.

The LAP also showed significant predictive ability, with an AUROC of 0.711 (95% CI: 0.584–0.838; p=0.002). The optimal cut-off for LAP was 71.2, resulting in a sensitivity of 71.2% and a specificity of 70.8%.

In contrast, both HSI and FSI showed poor diagnostic accuracy, yielding indeterminate results. The HSI had an AUROC of 0.525 (p=0.765), while the FSI had an AUROC of 0.536 (p=0.987). These results indicate that both indices have little discriminatory power for diagnosing NAFLD in our cohort.

We also evaluated the composite indices that integrate the TyG index with various anthropometric measures: TyG-WHtR, TyG-BMI and TyG-WC. The TyG-WHtR had an AUROC of 0.752 (95% CI: 0.626–0.878; p<0.001), indicating good diagnostic ability. The TyG-WC showed a slightly lower AUROC of 0.720 (95% CI: 0.588–0.852; p=0.002), while the TyG-BMI had the lowest AUROC among the composite indices at 0.638 (95% CI: 0.501–0.776; p=0.048), although it remained statistically significant.

Compared with previous studies, such as the one conducted in a Chinese population, our results for the TyG index align well, indicating its strong utility in screening for NAFLD across different populations.^10^ However, while TyG-WHtR and TyG-WC also demonstrated good diagnostic ability, they did not outperform the TyG index alone in our cohort. This contrasts with the findings from the Chinese study, where TyG-WC had the highest AUROC, outperforming the standalone TyG index.

A study conducted to determine the effectiveness of LAP in diagnosing NAFLD in obese children found that LAP had an AUROC of 0.698 (p=0.002).^11^

Another study conducted in an Italian population aimed to diagnose NAFLD with the help of different serum biomarker panels. The study found an AUROC of 0.84 (sensitivity 87% and specificity 64%) for the fatty liver index, an AUROC of 0.81 (sensitivity 93% and specificity 92%) for HSI, an AUROC of 0.86–0.87 (sensitivity 86% and specificity 71%) for the NAFLD liver fat score, an AUROC of 0.79–0.80 (sensitivity 85–100% and specificity 83–100%) for SteatoTest and an AUROC of 0.87 (sensitivity 92% and specificity 90%) for the NAFLD ridge score.^12^

There are many other scores and biomarker panels that have been used and are being developed to aid in the diagnosis of NAFLD, which were not covered in this study. One such score is the body mass index, AST/ALT ratio and diabetes score (BARD score), which has an AUROC of 0.808, while an enhanced version, the BARD INR score, has an AUROC of 0.881.^13^ Another test specific for fibrotic NAFLD is the homeostasis model assessment, AST and cytokeratin-18 (MACK-3) test, with an AUROC of 0.847 ± 0.030 (p≤0.002), a sensitivity of 90.0% and a specificity of 94.2% in a European population.^14^

The BARD and NAFLD-fibrosis scores are designed to predict the risk of advanced fibrosis in patients with NAFLD and are not intended for diagnosing or screening NAFLD. Therefore, they were not included in the study. MACK-3, on the other hand, is a diagnostic tool, but it is complex and requires multiple patient data points such as cytokeratin-18 and homeostatic model assessment for insulin resistance, as well as a calculator. Hence, it is not useful for screening all patients in an outpatient department setting and should be reserved for specific situations like screening for a randomized controlled trial.

Despite the availability of serum biomarker panels, liver biopsy remains the gold standard for the diagnosis of NAFLD. The main reason for pursuing the development of a good outpatient-based serum biomarker panel is to eliminate the need for an invasive and often stressful test for patients and to be able to safely diagnose NAFLD, aid in drug development and guide personalized treatment for the same.

Some other non-i nvasive modalities include USG, which is suggested as a first-l ine tool to define steatosis. Along with the exclusion of other causes of steatosis and chronic liver disease, it can help diagnose NAFLD, a crucial step in the management of the disease. This includes excluding fibrosis by means of scores, like the NAFLD-fibrosis score and the FIB-4 score, or performing transient elastography.^15^

MRI is another modality that has shown great promise in classifying grades and changes in hepatic steatosis. The controlled attenuation parameter (CAP) is a technique that helps measure liver stiffness and steatosis. Raised CAP values have been found to be associated with steatosis and metabolic syndrome. Other methods like NASH test and enhanced liver fibrosis panels are used for diagnosing NASH and fibrosis, respectively.^16^

Various genetic biomarkers are also being investigated for diagnosing NAFLD, such as genetic variations of PNPLA3 and TM6SF2. Other novel biomarkers, including bile acid biomarkers, multiple plasma lipid species across the NAFLD spectrum of disorders, circulating extracellular vesicles containing messenger RNA, proteins and microRNAs, and many microbiota-related biomarkers have been identified. The development of modern mass spectroscopy and other highly efficient technologies has played a big role in the development of panels that can include such novel biomarkers.^16^

This study highlights the high prevalence of NAFLD in the population of Indian women with morbid obesity. It emphasizes the potential of LAP as a useful tool for NAFLD screening, underlining its sensitivity and specificity. The present study is the first one to validate the LAP score in the Indian subcontinent. Thus, the outcomes obtained from this study provide support for the integration of LAP as an effective screening tool for NAFLD in the Indian population and underscores the significance of LAP as a reliable biomarker for NAFLD. This study also stands as a significant validation of non-i nvasive biomarkers’ effectiveness as a practical and economically viable screening approach for NAFLD, thus enhancing their utility as an accessible and cost-effective diagnostic modality.

Given that this investigation was conducted in a hospital setting, it is imperative to exercise prudence when generalizing the results to a broader population of individuals characterized by severe obesity. Our study highlights the promising utility of the TyG index as a screening tool. However, we acknowledge that the study’s sample size may have limited its statistical power to detect smaller effect sizes. Future studies are needed to strengthen the evidence for its use in clinical practice. Due to practical considerations, USG was employed as the diagnostic method for NAFLD instead of histological testing, perhaps resulting in a negligible impact on the accuracy of diagnosis. Additionally, the inevitable overlap of polycystic ovary syndrome, metabolic syndrome and prediabetes presents practical difficulties due to the multifactorial causation of morbid obesity.

Nonetheless, TyG index has shown promise in diagnosing NAFLD, allowing timely interventions and reducing complications. More research is needed for the validation and optimization of screening strategies, aiming to reduce the burden of NAFLD and improve health outcomes.

## Conclusion

NAFLD is prevalent among women with morbid obesity in southern India. The TyG index demonstrates high accuracy for screening NAFLD, with an AUROC of 0.835, a sensitivity of 95.1% and a specificity of 70.8%, making it a reliable non-i nvasive tool. The LAP also showed utility, with an AUROC of 0.711 at a cut-off of 71.2.

In conclusion, the TyG index appears to be the most promising tool for identifying NAFLD in our cohort, with LAP also showing potential as a secondary option. However, further research is needed to validate these findings and to determine the best screening and diagnostic strategies for NAFLD in this population.
